# Role of Vitamin D in the Metabolic Syndrome

**DOI:** 10.3390/nu13030830

**Published:** 2021-03-03

**Authors:** Lucía Melguizo-Rodríguez, Víctor J. Costela-Ruiz, Enrique García-Recio, Elvira De Luna-Bertos, Concepción Ruiz, Rebeca Illescas-Montes

**Affiliations:** 1 Biomedical Group (BIO277), Department of Nursing, Faculty of Health Sciences, University of Granada, 18016 Granada, Spain; luciamr@ugr.es (L.M.-R.); vircoss@ugr.es (V.J.C.-R.); enri.recio@gmail.com (E.G.-R.); elviradlb@ugr.es (E.D.L.-B.); rebecaim@ugr.es (R.I.-M.); 2 Instituto Investigación Biosanitaria, ibs.Granada, 18012 Granada, Spain; 3 Institute of Neuroscience, University of Granada, 18016 Granada, Spain

**Keywords:** metabolic syndrome, vitamin D, cardiovascular risk, diabetes mellitus, obesity, insulin resistance

## Abstract

The prevalence of hypovitaminosis D has risen in developed countries over the past few years in association with lifestyle changes and an increase in unhealthy habits. Vitamin D deficiency has been implicated in various diseases, including metabolic syndrome (MetS), which is clinically defined by a set of metabolic and vascular disorders. The objective of this study was to review scientific evidence on the relationship between MetS and vitamin D deficiency to support the development of prevention strategies and health education programs. An inverse relationship has been reported between plasma vitamin D concentrations and the features that define MetS, i.e., elevated serum concentrations of glucose, total cholesterol, low-density lipoproteins, triglycerides, glycosylated hemoglobin, and a high body mass index. Numerous studies have described the benefits of vitamin D supplementation to improve outcomes in individuals with MetS. Interventions to maintain optimal vitamin D concentrations are proposed as a preventive strategy against MetS.

## 1. Introduction

Metabolic syndrome (MetS) is characterized by the combination of central (intraabdominal) obesity, hypertension (HT), hyperglycemia, elevated serum levels of triglycerides and cholesterol, reduced levels of high-density lipoproteins (HDLs), and insulin resistance (IR). However, the definition and treatment of this syndrome remain controversial [1,2,3]. MetS is associated with a higher risk of developing cardiovascular disease and type 2 diabetes mellitus, and one of the variables most widely cited by clinicians to explain the etiopathology of this syndrome is IR.

The prevalence of MetS has increased over the past few years, which has been attributed not only to the aging of populations but also to the increase in obesity rates associated with lifestyle changes, including less healthy eating habits and lower physical activity levels. MetS is now considered a pandemic in developed countries and a source of major public health concern [4,5,6,7].

Researchers have proposed that reduced serum vitamin D levels are related to an increased risk of MetS [8]. The objective of the present study was to examine available scientific evidence on the relationship of vitamin D deficiency with the onset and development of MetS. These data will support the development of effective public health strategies to prevent MetS and promote a healthy lifestyle.

## 2. Metabolic Syndrome

MetS is a set of metabolic conditions that increases the risk of cardiovascular disease and diabetes. The components of MetS have been defined in various guidelines and consensus agreements [1,2,3] and currently comprise central (intraabdominal) obesity, HT, IR, and dyslipidemia [9].

The prevalence of MetS is estimated to be 25% worldwide [10], varying widely as a function of sex, age, and ethnicity [11,12]. A marked increase has been observed over recent years in its prevalence among young adults. Due to this situation, the control of HDL levels has been proposed as a preventive measure against MetS to reduce the incidence of associated diseases [6].

The etiologies for MetS are overweight/obesity, lack of physical activity, and genetic predisposition. The accumulation and dysfunction of adipose tissue result in IR, which plays a decisive role in MetS development. The distribution of adipose tissue is also considered an important factor, and the abdominal localization of excess adipose tissue has been most closely associated with IR. The consequent microvascular damage can give rise to vessel wall inflammation and HT, components of MetS [13].

## 3. Vitamin D

**Figure 1:**
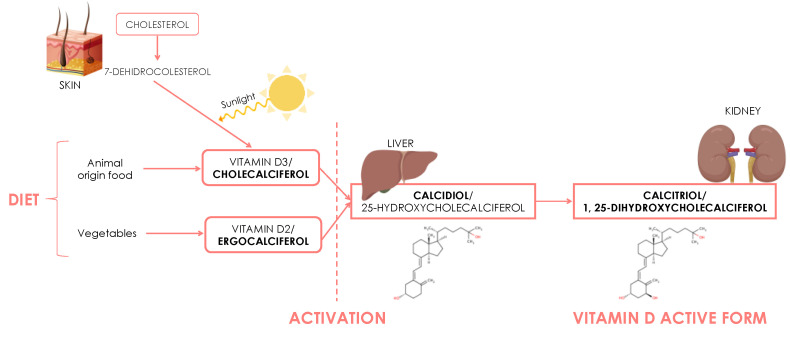
Vitamin D synthesis pathways.

Vitamin D is a fat-soluble prohormone that plays an essential role in bone mineral metabolism, being involved in calcium and phosphorus metabolism and skeletal homeostasis. The main source of vitamin D is cholecalciferol or vitamin D3, synthesized by sunlight on the skin from 7-dehydrocholesterol, for which cholesterol is a precursor [14]. It is also available in the diet from animal (cholecalciferol) and vegetable (ergocalciferol) foods. Regardless of the source, vitamin D requires two hydroxylations in the organism to become biologically active, the first in the liver and the second in the kidney, resulting in the form known as 1,25(OH)_2_ vitamin D or calcitriol (Figure 1) [15].

In the past, vitamin D was almost exclusively associated with bone health; however, numerous other functions have gradually emerged, and deficiency of this vitamin has been associated with a higher risk of certain autoimmune diseases [16]. These extraskeletal actions are enabled by the presence of vitamin D receptors and hydroxylation enzymes in the cells of different human tissues and by differences in vitamin D production depending on the tissue in which it is expressed. Therefore, the fact that the vitamin D receptor and the enzyme 1α-hydroxylase are expressed in different tissues (kidney, pancreas, prostate, or immune system) shows the possible action of this vitamin on these tissues. Thus, changes in the expression of vitamin D receptors may be associated with the development of MetS and its different components [17,18,19]. Vitamin D has also been attributed to hormonal activity, including endocrine, autocrine, and paracrine functions [8]. In addition to all these activities, vitamin D has other functions of pleiotropic nature such as its anti-inflammatory, anti-apoptotic and anti-fibrotic effects, preventive action against cardiovascular and renal diseases, diabetes mellitus, or cancer through different mechanisms of action widely described [20].

Interest in this vitamin has intensified over recent years due to the high prevalence of hypovitaminosis D, described as a worldwide epidemic [21]. Based on the levels of vitamin D, we can talk about insufficiency when 25(OH)D of 21–29 ng/mL, mild deficiency when levels are between 10 and 20 ng/mL, moderate deficiency between 9 and 5 ng/mL, and severe deficiency when vitamin D levels are lower than 5 ng/mL [22,23,24]. The very high percentage of individuals with hypovitaminosis D has highlighted the need to implement preventive strategies [25].

## 4. MetS and Vitamin D Deficiency

Numerous authors have addressed the possible association between micronutrient deficiency and metabolic disorders, including MetS [25]. Various pathophysiologic mechanisms have been proposed to underlie the effect of vitamin D on MetS components. One plausible explanation is that vitamin D affects insulin secretion and sensitivity, which play a key role in MetS development. The vitamin D receptor is expressed by β cells in the pancreas and in musculoskeletal and adipose tissues, among other peripheral tissues, and vitamin D deficiency can compromise the capacity of β cells to convert pro-insulin into insulin [26]. Another pathophysiological mechanism could be related to the association between obesity and vitamin D deficiency. The two most accepted hypotheses are vitamin D sequestration and volumetric dilution. In the first case, this vitamin is sequestered in adipose tissue, increased in obese individuals, which also influences a greater volumetric dilution, according to which 25(OH)D, a fat-soluble molecule, would be distributed among fat, muscle, liver, and serum, decreasing serum vitamin D levels. Other possible explanations for this relationship described may be poor dietary habits, decreased sun exposure, the difference in gene expression in vitamin D metabolizing enzymes, and impaired hepatic 25-hydroxylation [27,28].

Serum concentrations of vitamin D vary widely among different geographic areas, largely attributable to differences in sun exposure, a key source of vitamin D [15]. Thus, the frequency of hypovitaminosis, and therefore the potential risk of developing diabetes or MetS is higher in populations living further from the equator, and differences in clothing habits, skin color, and the use of sunscreen also play a role [29]. With regard to specific populations, vitamin D deficiency in postmenopausal women seems to be associated with a higher risk of MetS, hypertriglyceridemia, and lower HDL levels [26]. As noted above, deficiency of this vitamin is more frequent in older age groups, with Navarro et al. reporting that levels were inadequate in 50% of individuals aged 18 to 60 years and in 87% in those aged over 65 years [30]. In this context, Xu et al. observed that those subjects with genetically increased 25(OH)D concentration were less at risk of type 2 diabetes [31].

A study of non-diabetic young people also showed an inverse relationship between vitamin D levels and the presence of MetS, attributed to the combined effect of obesity and IR [32]. Lee et al. described a higher risk of MetS in Korean men and women aged over 65 years with low 25(OH)D levels. After adjusting for age, area of residence, season, and habits (exercise, tobacco, and alcohol), there appeared a relationship between low vitamin D levels and increased prevalence of MetS, so the lower the vitamin D levels (14.20–18.99 ng/mL in men and 11.20–15.59 ng/mL in women) the higher the prevalence of elevated waist circumference, hypertriglyceridemia, and high low-density lipoprotein cholesterol (LDL) concentrations [33]. Likewise, Zhu and Heil reported that 25(OH)D levels were inadequate in 50% of the study population, formed by residents of Shanghai, China, aged 19–70 years and were associated with the presence of MetS, observing a linear relationship between 25(OH)D concentrations and serum concentrations of glucose and lipids [34]. They calculated that each increase of 1 ng/mL 25(OH)D was associated with a significant reduction in total cholesterol and LDL and a 54% decrease in the risk of MetS. Subsequent studies also reinforce the possible association between vitamin D deficiency and MetS prevalence [35,36,37].

Barbalho et al. found that 80% of patients in a cardiology unit had vitamin D deficiency and that all of the patients with hypovitaminosis D had MetS. They also observed significantly higher levels of glycemia, glycosylated hemoglobin, total cholesterol, LDLs, triglycerides, and atherogenic indices and an elevated body mass index in patients with vitamin D deficiency in comparison to those with adequate vitamin D levels [38]. Vimaleswaran et al., meanwhile, noted that increased plasma concentrations of 25(OH)D might reduce the risk of hypertension. Each 25(OH)D-increasing allele of the synthesis score was associated with a change of −0.10 mm Hg in systolic blood pressure and a change of −0.08 mm Hg in diastolic blood pressure [39]. However, some authors question or discuss the association between vitamin D deficiency and components associated with MetS [40,41,42]. Likewise, Mehri et al. pointed out that the absence of a long follow-up meant that a causative relationship could not be definitively established between inadequate vitamin D levels and MetS [43]. Teixeira et al. considered vitamin D deficiency to be secondary to the metabolic changes in MetS, although it was associated with IR, which is closely related to the development of MetS [44]. Similarly, Chen et al. described that the Mendelian randomization (MR)-derived odds ratio of genetically determined 25(OH) D for risk of MetS was 0.977, and therefore, it cannot be concluded that genetically conditioned reduction in vitamin D levels can increase the risk of metabolic syndrome or any of its components [45]. All of these authors call for further research to elucidate this relationship.

In summary, the relationship between vitamin D deficiency and the components of MetS remains a controversial issue because no conclusive scientific evidence is available. Thus, the main findings of the previously cited studies are summarized in Table 1.

## 5. Effect of Vitamin D Supplementation on MetS

Vitamin D supplementation has been reported to exert a beneficial effect in the treatment of MetS-related diseases, such as lipid profile, insulin resistance and hyperglycemia, obesity, and hypertension [46] (Table 2). This effect could be based on the mechanism of action of vitamin D on different physiological parameters, including improved arterial stiffness; decreased renin-angiotensin-aldosterone system activity, parathyroid hormone levels, inflammatory cytokines; increased activity of lipoprotein lipase; and improved phospholipid metabolism and mitochondrial oxidation [47].

### 5.1. Vitamin D Supplementation and Insulin Resistance and Hyperglycemia

In relation to IR, several studies have shown positive effects of vitamin D supplementation in pre-diabetic patients [48,49]. In their work, Lemieux et al. demonstrated that supplementation with 5000 IU daily of vitamin D for 6 months increased insulin sensitivity and pancreatic β-cell activity [48]. Other authors showed the benefits of supplementation with 50,000 IU/week of vitamin D for 8 weeks in patients with type 2 diabetes, with a reduction in glycosylated hemoglobin and an increase in sirtuin 1, which appears to be related to increased insulin secretion by pancreatic β-cells [50]. In their study on diabetic patients, Farrokhian et al. also observed that an administration pattern of 50,000 IU every 2 weeks for 6 months reduces basal glycemia, increasing insulin sensitivity [51]. Furthermore, in this population, co-supplementation with vitamin D (50,000 IU, twice a week for six months) and omega-3 fatty acids has also been found to produce a significant decrease in fasting blood glucose levels, increasing insulin sensitivity [52]. However, other studies seem to show that vitamin D administration has no effect on insulin resistance in pre-diabetic patients [53]. Lerchbaum et al. further claimed that vitamin D treatment might have a negative effect on insulin sensitivity in healthy men [54].

### 5.2. Vitamin D Supplementation and Dyslipidemia

According to the scientific literature, vitamin D supplementation may also exert a beneficial effect on the lipid profile. In this regard, Gadheri et al. showed a significant reduction in triglyceride and low-density lipoprotein cholesterol (LDL) levels after supplementation with 50,000 IU of vitamin D every 2 weeks for 3 months [55]. Jamilian et al., who used the same doses for 6 months, observed improvements in triglycerides and very-low-density lipoprotein cholesterol [56]. Other administration patterns have also been effective in reducing plasma lipid levels, such as those proposed by Riek et al. or Imga et al., which led to a decrease in LDL [57,58], or that proposed by Liyanage et al., who suggested that parenteral administration of vitamin D leads to an increase in plasma high-density lipoprotein cholesterol (HDL) levels [59]. The favorable effects of this vitamin on HDL described above have been corroborated by the meta-analysis developed by Ostadmohammadi et al. [60]. Furthermore, these changes in lipid profile have also been found in children with type I diabetes [61]. However, Farrokhian et al. found no significant changes in the lipid profile following vitamin D administration, although they did observe changes in plasma malonaldehyde levels, which results from lipid peroxidation [51], results that were later corroborated by Tamadon et al. [62].

### 5.3. Vitamin D Supplementation and Obesity

With regard to the effects on obesity, according to two meta-analyses recently published, it has been shown that vitamin D supplementation could contribute to reduced body mass index (BMI) and waist circumference, but not weight loss [63]. In this line, a meta-analysis of 22 observational studies determined that despite an inverse relationship between the percentage of fat mass and serum vitamin D levels, vitamin D supplementation was not found to significantly decrease the percentage of fat mass with respect to placebo groups [64]. In contrast, Lotfi-Dizaji et al. observed a decrease in weight and fat mass in those subjects with vitamin D deficiency who took 50,000 IU of vitamin D for 12 weeks [65]. However, there are studies showing contrary results, finding no beneficial effect of vitamin D supplementation at the level of parameters such as BMI, weight, hip circumference, or fat percentage [66,67,68,69]. Similarly, recent studies in individuals aged 6–14 years showed that vitamin D supplementation did not demonstrate any effect on BMI, waist circumference, waist-to-hip ratio, and percentage of fat tissue [70,71,72]. Nevertheless, the combined action of calcium supplementation together with vitamin D3 appears to increase weight loss and improve some of the blood metabolic profiles in obese women [66].

### 5.4. Vitamin D Supplementation and Hypertension

A significant reduction in blood pressure has been observed following the administration of vitamin D [73,74], although Golzarand et al. observed that in some cases, this supplementation can cause hypotension in both healthy and hypertensive subjects [73]. However, further analyses performed on randomized clinical trials that aimed to study the effect of such supplementation on blood pressure (BP) reported that there is no significant effect on systolic (SBP) or diastolic (DBP) blood pressure values [75,76,77]. Similar results are obtained by studying supplementation in non-adult populations [78]. However, a recently published clinical trial not included in the previous review indicates that after 6 months of supplemental treatment at the dose of 1000 IU daily could decrease SBP and DBP figures [70]. Whenever the effect of combined vitamin D and calcium supplementation on BP is analyzed, the results are very heterogeneous; whereas some authors find no significant effect on SBP and a reduction of DBP [79], others observed an elevation of SBP and DBP [73].

### 5.5. Vitamin D Supplementation Dosage

No consensus has been reached on the optimal treatment of vitamin D deficiency. Various regimens of daily or monthly doses of cholecalciferol have all obtained suitable outcomes. Carbonare et al. found that 80% of patients receiving 1750 IU/day or 50,000 IU/month supplementation for six months reached serum 25(OH)D levels > 30 ng/mL [80]. Optimal vitamin D levels are clearly beneficial for metabolic and cardiovascular health in general [15,81]. Various authors have emphasized the need for supplementation to maintain adequate vitamin D levels and thereby reduce the risk of MetS and associated diseases [46,82]. Therefore, it appears essential to determine the vitamin D levels of populations for the development of supplementation interventions and to implement public health programs on healthy habits that prevent vitamin D deficiency.

## 6. Conclusions

According to the different studies analyzed in this review, vitamin D deficiency appears to be associated with the different components that define MetS. Similarly, vitamin D supplementation may be an appropriate strategy in the treatment of MetS. However, it is not possible to draw a clear conclusion on this association as the published data are contradictory and it is not clear whether vitamin D deficiency is a cause or effect of the metabolic syndrome or any of its components. Therefore, further studies are needed to determine the real role of vitamin D deficiency in the development of MetS.

## Figures and Tables

**Table 1:** Key findings of reviewed studies.

Ref.	Year	MetS and Vit D	Objective	Methodology	Findings
Valero et al. [15]	2007	Endogenous and exogenous sources of vitamin D and its role in metabolism.	To investigate the sources where they can take vitamin D from requirements, intake, and effects on health.	Review	Physiological levels of 25(OH)D are required to keep the integrity of immune, bone, and muscular systems. Sunlight exposure is not enough for reaching and keeping acceptable levels of Vit D in some age groups. Artificial addition of Vit D in food has showed its efficacy for reaching these desirable levels, and most of the population would profit from it. For people over 65, calcium addition is also necessary.
Mansouri et al. [25]	2018	Nutrient deficit and metabolic disorders.	To determine the link between vitamin D and metabolic syndrome.	Cross-sectional study. A total of 352 faculty members. Blood samples for the determination of 25(OH)D concentrations, glycemic indicators, and lipid profile.	Reverse association of 25(OH)D serum levels and risk of abdominal obesity, hypertension, and abnormal glucose homeostasis. No significant association for metabolic syndrome.
Schmitt et al. [26]	2018	Vitamin D-deficiency and metabolic syndrome in postmenopausal women.	To study vitamin D-deficiency and its association with risk factors for metabolic syndrome in postmenopausal women.	Observational and cross-sectional cohort study. A total of 463 women. Levels of total cholesterol, HDL, LDL, triglycerides, glucose, insulin, and 25(OH)D were measured.	Deficiency of vitamin D in postmenopausal women is related to a higher prevalence of MetS, as well as hypertriglyceridemia and low HDL levels.
Wimalawansa et al. [28]	2018	Association between vitamin D, insulin resistance, obesity, type II diabetes, and metabolic syndrome.	To investigate the relationship between vitamin D and insulin resistance, obesity, type II diabetes, and metabolic syndrome.	Review	Large number of observational studies point out the improvement of type II diabetes, insulin resistance, obesity, and metabolic syndrome with adequate levels of vitamin D.
Mutt et al. [29]	2019	25(OH)D levels and MetS in the elderly population from Northern latitudes.	To investigate the associations between serum 25(OH)D levels and prevalence of MetS and its components and assess the effects of vitamin D supplementation on MetS.	Cross-sectional study. A total of 636 subjects from Oulu45 cohort (263 male, 373 female). Determination of 25(OH)D plasmatic levels and assessment of vitamin D supplements usage.	Low vitamin D levels were associated with a higher prevalence of MetS. People under vitamin D supplementation had a lower incidence of MetS and its components. Low vitamin D levels are a risk factor for MetS among other lifestyle factors among older subjects in the Northern latitudes.
Navarro et al. [30]	2014	Status of vitamin D levels in the Spanish population.	To determine the levels of vitamin D in Spain.	Review	There is an insufficiency of vitamin D in the Spanish population. There are 50% of the population between 18 and 60 years with this deficiency, and up to 87% in the population over 65 years old.
Xu et al. [31]	2020	Genetically increased circulating 25(OH)D level and prevention of T2D.	To provide an updated estimate for the causality between vitamin D and T2D.	2-sample multi-instrument variables MR	A higher genetically instrumented 25(OH)D was causally linked to a reduced risk of T2D risk. They confirm the causal role of vitamin D using 2 synthesis-related single-nucleotide polymorphisms (SNPs).
Huang et al. [32]	2015	Vitamin D and risk of metabolic syndrome.	To analyze vitamin D levels and the link with the risk of metabolic syndrome in non-diabetic adults.	Cross-sectional study. A total of 335 non-diabetic young adult individuals. Measurement of 25(OH)D, metabolic syndrome, and cardiometabolic parameters.	There is an inverse association between vitamin D and MetS. This link could be related to the joint effects of obesity and insulin resistance in individuals.
Lee et al. [33]	2019	25(OH)D levels and MetS in the elderly population.	To evaluate the relationship between 25(OH)D levels and MetS in the elderly Korean urban and rural population.	Cohort study. A total of 2936 men and women. Measurement of 25(OH)D serum levels as well as diagnosis of MetS.	There is an association between low levels of 25(OH)D and MetS. Those levels achieved more association in these variables of MetS: high waist circumference, hypertriglyceridemia, as well as low high-density lipoprotein cholesterol.
Zhu et al. [34]	2018	Vitamin D and markers of metabolic health.	To investigate the link between vitamin D and markers of metabolic health.	Cross-sectional study. A total of 508 urban residents. Measurement of demographic and anthropometric data, as well as 25(OH)D serum levels, blood glucose, and lipid concentrations.	A higher serum 25(OH)D concentration was linked to a better metabolic profile and less risk for developing MetS.
Liu et al. [35]	2020	Vitamin D-deficiency and MetS criteria in the elderly population.	To analyze the association between serum 25(OH)D and MetS in elderly Chinese individuals.	Cross-sectional study. A total of 2493 elderly people from eight areas of China. 25(OH)D serum levels as well as antropometric and biochemical measurements were determined	High serum vitamin D concentrations were associated with a low prevalence of MetS according to the Adult Treatment Panel III criteria for adequate versus deficient vitamin D and inadequate versus deficient vitamin D levels.
Ganji et al. [36]	2020	Vitamin D deficiency and MetS prevalence and markers.	To study the relationship between serum vitamin D concentrations and prevalence of MetS and markers of MetS in Qatari women.	Cross-sectional study. A total of 700 women aged 20–80 years old. Independent variable:serum 25(OH)D concentration-dependent variables:MetS and indicators of MetS defined following the International Diabetes Federation criteria	The study showed an inverse relationship between the prevalence of MetS and serum 25(OH)D in Qatari women. No relationship was observed between serum 25(OH)D and waist circumference, blood pressure, HbA1C, blood glucose, HDL-cholesterol, and serum triglycerides.
Pott-Junior et al. [37]	2020	MetS parameters and low serum 25-hydroxyvitamin D (25(OH)D) levels.	To investigate the relationship between metabolic parameters and serum 25(OH)D levels in community-living older adults.	Cross-sectional study. *n* = 265. Adults aged 60 years were assessed for anthropometrics and metabolic measurements, including 25(OH)D, insulin, glucose, total cholesterol, high-density lipoprotein cholesterol, low-density lipoprotein cholesterol, triglycerides, and inflammatory markers.	Subjects with 25(OH)D deficiency presented higher body weight, body mass index, waist circumference, triglycerides and TNF-α, and higher insulin resistance. MetS was more prevalent among 25(OH)D-deficient subjects.
Barbalho et al. [38]	2018	Vitamin D and markers of metabolic health.	To investigate the link between vitamin D and markers of metabolic health.	Cross-sectional study. A total of 200 patients (89 men, 111 women). Determination of anthropometric and biochemical parameters, blood pressure, atherogenic indices, and presence of MetS.	Patients with altered values for this vitamin presented significantly higher values for glycemia, HbA1c, total cholesterol, LDL-c, triglycerides, BMI, waist circumference, and atherogenic indices.
Vimaleswaran et al. [39]		Vitamin D and arterial blood pressure and hypertension risk.	To test whether 25(OH)D concentration is causally associated with blood pressure and hypertension risk.	MR study	Increased plasma concentrations of 25(OH)D might reduce the risk of hypertension. Each 25(OH)D-increasing allele of the synthesis score was associated with a change of −0.10 mm Hg in systolic blood pressure and a change of −0.08 mm Hg in diastolic blood pressure.
Vimaleswaran et al. [40]		Vitamin D status and obesity.	To explore the causality and direction of the relationship between body mass index (BMI) and 25-hydroxyvitamin D [25(OH)D].	Bidirectional MR study	Higher BMI leads to lower 25(OH)D, whereas any effects of lower 25(OH)D-increasing BMI are likely to be small.
Zheng et al. [41]		Circulating 25-hydroxyvitamin D metabolites and T2D.	To examine the potential causality of these associations using Mendelian randomization (MR) analysis.	MR study	The findings based on MR analysis in a large sample of European ancestry do not support a causal association of a total of 25(OH)D or 25(OH)D metabolites with T2D and argue against the use of vitamin D supplementation for the prevention of T2D.
Wang et al. [42]		Vitamin D, prediabetes, and T2D.	To explore the causal relationship between 25-hydroxyvitamin D (25(OH)D) and glycemic status and indices.	Biredictional MR	The MR-derived odds ratios of genetically determined 25(OH)D for risk of T2D and prediabetes were 0.985 and 0.982, respectively. Fasting glucose and HbA1c were not significant either.
Mehri et al. [43]	2019	Vitamin D and MetS and its components in females.	To determine the relationship between MetS and its components with vitamin D status.	Observational case-control study. Participants were 276 Iranian female teachers (124 in the case group and 152 in the control group).	Authors did not find an association between vitamin D status and MetS. There is a necessity of using prospective studies to link the vitamin D effects in the development of MetS.
Teixeira et al. [44]	2018	Association between vitamin D-deficiency and obesity.	To analyze vitamin D and metabolic profile in adolescents and adults and their relationship with severe obesity complications.	Observational comparative study. Population: adolescents and adults with severe obesity. Measurement of circumference and BMI.	There was a high prevalence of deficiency and insufficiency of vitamin D and its association with metabolic changes in the adult and adolescent population with obesity.
Chen et al. [45]		25-hydroxyvitamin D cardiometabolic risk factors and MetS.	To test whether genetically lowered vitamin D levels were associated with MS and its metabolic traits.	MR study	Lower measured 25(OH)D levels were associated with MetS after multivariable adjustment. However, the MR-derived odds ratio of genetically determined 25(OH) D for risk of MS was 0.977.

MetS: metabolic syndrome; BMI: body mass index; LDL: low-density lipoprotein cholesterol; HDL: high-density lipoprotein cholesterol; HbA1c: hemoglobin A1c; TNF-α: tumor necrosis factor-α; MR: Medelian randomization; T2D: type 2 diabetes.

**Table 2:** Summary of the effects of vitamin D supplementation on metabolic syndrome.

Ref.	Year	Supplementation Characteristics	Findings
Lemieux et al. [48]	2019	Vitamin D3 5000 IU daily, 6 months	In pre-diabetic patients, vitamin D supplementation for 6 months significantly increased peripheral insulin sensitivity and β-cell function.
Babarawi et al. [49]	2020		In patients with prediabetes, vitamin D supplementation at moderate to high doses (≥1000 IU/day) significantly reduced the incidence risk of T2DM compared with placebo.
Safarpour et al. [50]	2020	50,000 IU/week vitamin D (Zahravi Co^®^ pearls), 8 weeks	Vitamin D supplementation improved T2D by decreasing HbA1c and increasing SIRT1.
Farrokhian et al. [51]	2017	50,000-IU vitamin D supplements every 2 weeks, 6 months	Compared with placebo, vitamin D supplementation resulted in significant reductions in fasting plasma glucose, serum insulin, homeostasis model assessment of insulin resistance, and β cell function.
Talari et al. [52]	2019	50,000 IU vitamin D supplements every 2 weeks + 2 × 1000 mg/day n-3 fatty acids from flaxseed oil, 6 months	Vitamin D and n-3 fatty acids’ co-supplementation led to a significant reduction in fasting plasma glucose, insulin, insulin resistance and LDL, and a significant increase in insulin sensitivity and HDL compared with the placebo.
Wallace et al. [53]	2019	3000 IU (75 µg) vitamin D3 daily, 26 weeks	There was no difference between treatment and placebo group in measures of whole-body, peripheral, or hepatic IR or in any measure of glycemic control or β-cell function.
Lerchbaum et al. [54]	2019	20,000 IU of vitamin D3/week, 12 weeks	In healthy middle-aged men, vitamin D treatment had a negative effect on insulin sensitivity.
Ghaderi et al. [55]	2017	50,000 IU vitamin D supplements every 2 weeks, 12 weeks	Patients who received vitamin D supplements had significantly decreased fasting plasma glucose, serum insulin levels, homeostasis model of assessment-insulin resistance, serum triglycerides total, and LDL compared with the placebo.
Jamilian et al. [56]	2017	50,000 IU vitamin D every 2 weeks + 1000 mg omega-3 fatty acids twice a day, 6 weeks	Overall, vitamin D and omega-3 fatty acids co-supplementation for 6 weeks among gestational diabetes patients had beneficial effects on fasting plasma glucose, serum insulin levels, the homeostatic model of assessment for insulin resistance, quantitative insulin sensitivity check index, serum triglycerides, and very-low-density lipoprotein cholesterol levels.
Imga et al. [57]	2019	100,000 IU/week as a loading dose for 8 weeks following a maintenance dose of 3000 IU/day	After the sixth month of supplementation in both overweight and obese subjects, a significant reduction was detected in HOMA-IR, low-density lipoprotein cholesterol, and parathyroid hormone levels.
Riek et al. [58]	2018	Vitamin D3 4000 IU/day for 4 months	Assessment of oxidized LDL uptake in monocytes cultured in the patient’s own serum before vs. after treatment resulted in >50% reduction in the vitamin D group with no change in the placebo group. The reduction in monocyte cholesterol uptake was reflected in a 19% decrease in total monocyte cholesterol content.
Liyanage et al. [59]	2017	50,000 IU of vitamin D3 intramuscularly, monthly for 6 months	There was a significant improvement of serum HDL level with six months of therapy of high-dose vitamin D in patients with early diabetic nephropathy.
Ostadmohammadi et al. [60]	2019		This meta-analysis demonstrated the beneficial effects of vitamin D supplementation on improving glycemic control, HDL, and C-reactive protein levels among patients with cardiovascular diseases.
Hafez et al. [61]	2019	VD3 in the form of 4000 IU/day for a period of 4 months	After 4 months of vitamin D supplementation, the mean difference (at 0 and 4 months) in LDL and HbA1c was statistically significant.
Tamadon et al. [62]	2018	oral vitamin D3 supplements at a dosage of 50,000 IU every 2 weeks for 12 weeks	No significant effect of vitamin D supplementation on lipid profiles and other biomarkers of inflammation and oxidative stress compared with the placebo was observed.
Perna [63]	2019	Vitamin D3 supplementation, from 25,000 to 600,000 IU/monthly of cholecalciferol. Duration of the treatment: from 1 to 12 months.	Cholecalciferol supplementation decreases the BMI by −0.32 kg/m^2^ and the waist circumference by −1.42 cm but does not statistically affect weight loss −0.43 kg.
Golzarand et al. [64]	2018	Supplementation from 400 to 7000 IU of daily cholecalciferol. Duration of the treatment: range from 3 to 12 months.	Cholecalciferol supplementation increased in individuals after the intervention. A decrease in body fat percentage was found (−0.31%, 95% CI: −1.07 to 0.44), although this was not significant.
Lotfi-Dizaji et al. [65]	2019	Weekly bolus dose of 50,000 UI od vitamin D. Duration of the treatment: 12 weeks.	An increase in serum 25(OH)D levels was found after vitamin D administration. The analysis showed a significant decrease in weight and body fat percentage after comparison between groups.
Subih et al. [66]	2020	Supplementation consisted of vitamin D3, with doses of 50,000 IU weekly, or vitamin D3 combined with calcium, with doses of 50,000 IU weekly of vitamin D and 1,200 mg/dL daily of calcium. Duration of the treatment: 3 months.	Compared to the control group, those women supplemented with vitamin D did not show significant changes related to weight loss or improvement of obesity biomarkers. However, after co-supplementation of calcium and vitamin D, a decrease in weight was reported, as well as an improvement in some of the blood metabolic profiles.
Duan et al. [67]	2018	Daily doses of vitamin D supplementation from 100 to 8571 UI. Duration of the treatment: range from 1.5 to 36 months.	Comparisons with the placebo or control group determined that neither BMI nor waist circumference decreased significantly after vitamin D administration.
Gallagher et al. [68]	2013	Daily doses of vitamin D ranged from 400 to 4800 IU. Duration of the treatment: 12 months.	Although an inverse relationship was found between serum vitamin D levels and fat mass, between-group analyses found no significant reductions in BMI or fat mass after supplementation.
Chandler et al. [69]	2015		When compared with placebo, vitamin D supplementation had no significant effect on BMI, weight, or fat mass. Likewise, no significant reduction in BMI, weight, or fat mass was observed in participants who received vitamin D plus calcium compared with those who received calcium control.
Rajakumar et al. [70]	2020	Daily vitamin D oral doses containing 600, 1000, and 2000 UI. Duration of the treatment: 6 months.	Vitamin D supplementation showed no effect on BMI, waist circumference, waist-to-hip ratio, and percentage of fat tissue in children. In contrast, a significant decrease in SBP and DBP levels was observed after daily administration of 1000 IU.
Brzeziński et al. [71]	2020	Daily supplementation of 1200 UI of vitamin D. Duration of the treatment: 26 weeks.	There showed no significant decrease in either BMI or weight of obese children in comparison with the placebo group.
Makariou et al. [72]	2020	The vitamin D administration regimen was 2000 IU daily for a duration of 3 months.	The results did not show a relationship between vitamin D supplementation and BMI, blood pressure, lipids, glucose, and insulin levels, among others.
Golzarand et al. [73]	2016	The dose of vitamin D administration ranged from 200 to 12,000 IU per day. The mean duration of treatment was 5.6 ± 4.0 months.	The results showed that the daily administration of vitamin D supplementation in doses higher than 800 IU in subjects older than 50 years significantly reduced both SBP and DBP (*p* < 0.001). Moreover, hypotensive effects were observed in both the group of healthy subjects and the group of hypertensive subjects.
Shu & Huang [74]	2018	The daily dose of vitamin D administered was from 2800 UI to more than 5000 UI. Duration of the treatment: from 8 to 20 weeks.	Results show a significant effect of vitamin D supplementation on peripheral DBP in vitamin D-deficient participants, but not the same with peripheral SBP. Subgroup analysis showed a significant decrease in peripheral SBP and DBP in Asia, 8 weeks of intervention, and more than 5000 IU of daily vitamin D supplementation subgroups.
Wu et al. [75]	2010	The vitamin D dose administered varied in a range between 200 IU and 400 UI per day. Duration of the treatment: from 5 to 15 weeks.	The analysis concluded that vitamin D supplementation reduced SBP by 2.44 mmHg, but no effect on DBP was observed compared to the placebo groups.
Hussin et al. [76]	2017	The daily vitamin D administration regimen varied between 1000 UI and 5000 UI. Treatment duration ranged from 4 to 52 weeks.	The effects of vitamin D supplementation showed no improvement in endothelial function. Specifically, no significant changes in blood pressure, post-occlusive vasodilation of the brachial artery, among others, were observed.
Swart et al. [77]	2018	Vitamin D supplementation varies between 200 IU and 7000 IU daily. The duration of treatment ranged from 8 weeks to 1 year.	All the studies analyzed found a significant increase in serum vitamin D levels. However, vitamin D supplementation had no effect on SBP and DBP.
Abboud et al. [78]	2020	Vitamin D administration regimens were in a daily range between 400 IU and 43,000 IU, with a duration between 12 and 24 weeks.	The results showed that vitamin D supplementation was ineffective in reducing SBP and DBP in children and adolescent populations.
Morvaridzadeh et al. [79]	2020	Vitamin D was administered daily between 200 IU and 7142 IU. Calcium dosage ranged from 500 to 1200 mg daily. Treatment duration ranged from 6 weeks to 7 years.	Vitamin D and calcium co-supplementation did not show a reduction in SBP compared to the control group, although a significant decrease in DBP was found.
Carbonare et al. [80]	2018	Vitamin D3	Two regimens were equivalent; most of the patients reached the normal range of vitamin D after six months of treatment with increased calcium levels and decreased bone turnover. Waist circumference also decreased. The relationship between serum 25(OH)vitamin D3 concentration and waist circumference supports vitamin D having a protective role in the current setting since waist size is directly associated with the risk of cardiovascular and metabolic diseases.
Al-Daghri et al. [82]	2019	Vitamin D3vitamin D-fortified milk	There was an increase in 25(OH)D levels in all groups. It is also revealed a clinically significant decrease in triglycerides, glucose, and systolic blood pressure, as well as a clinically significant increase in HDL over time, all in favor of the Vit D group. MetS incidence did decrease in the Vit. D group only. Consequently, oral vitamin D supplementation is superior to vitamin D-fortified milk in improving vitamin D status. Reduction in the incidence of MetS in the Arab adolescent population secondary to vitamin D correction may be dose-dependent.

HbA1c: hemoglobin A1c; BMI: body mass index; DBP: diastolic blood pressure; SBP: systolic blood pressure; HDL: high-density lipoprotein cholesterol; LDL: low-density lipoprotein cholesterol; T2DM: type 2 diabetes mellitus; HOMA-IR: Homeostatic Model Assessment for Insulin Resistance.

## Data Availability

No new data were created or analyzed in this study. Data sharing is not applicable to this article.
